# Supplementary material for: A public generalizable AI tool for automated segmentation of coronal brain tissue slabs for 3D neuropathology
Source: PLoS One. 2026 Aug 17;21(8):e0355740. doi: 10.1371/journal.pone.0355740 (PMC13480592; doi:10.1371/journal.pone.0355740)
Supplement: Supplementary file 1 [file pone.0355740.s007.pdf]

Athinoula A.
Martinos
Center
For Biomedical Imaging

June 15, 2026

Dear Dr. Giove,

We would like to thank the editor and reviewers for their time and insightful feedback on our submission "A public generalizable AI tool for automated segmentation of coronal brain tissue slabs for 3D neuropathology." We have carefully reviewed all comments and made appropriate changes to our manuscript.

Specifically, we have revised the manuscript to provide a clearer and more comprehensive account of the study's methodology, context, and practical implications. We expanded the discussion of the image processing workflow and open-source tools, strengthened the manuscript's connection to prior work on segmentation methods, and clarified how our model may be applied to neuropathology image analysis. We also added further evaluation of model performance in challenging out-of-distribution cases, including visual examples of disagreement between predictions and manual annotations. Together, these revisions improve the manuscript's technical transparency, situate the work more fully within the existing literature, and provide clearer guidance for future development and end-user application.

Attached are our responses to all comments point-by-point, additionally to a revised manuscript with all changes highlighted in red.

Sincerely,

Jonathan Williams Ramirez, on behalf of the authors

The Athinoula A. Martinos Center for Biomedical Imaging @ Massachusetts General Hospital
149 13th Street, Suite 2301 | Charlestown, MA 02129

Response to Reviewer 1

Comment 1: *Elaborate on Generalization and Site Diversity*
While the model demonstrates strong robustness on out-of-distribution data, you correctly identify that data diversity is a limitation, as training and evaluation involved only three sites.
Suggestion: It would be beneficial to expand the discussion on how the tool might perform in more "extreme" out-of-distribution scenarios, such as varying lighting conditions, non-standard backgrounds, or different camera angles. Additionally, explicitly recommending future external validation by independent institutions with different photographic protocols would provide a clearer roadmap for the tool's adoption.

We included a more in-depth discussion as to how different aspects of photography setups may affect performance of the segmentation tool. We expanded the discussion session to discuss scenarios for out of distribution data, as well as outlined recommendations for data acquisition for prospective data – to get the most performance out of the segmentation model. We have modified the following text from the discussion

> While the diversity in dissection protocols, imaging setups, and tissue conditions (e.g., fixed vs. fresh) helps build robustness, it is still limited in scope. For best performance we recommend for users to use a high-contrast black background, with an overhead camera angle perpendicular to the dissection table. Because of variability lighting conditions in the training data, we expect that the tool will be more resistant to lighting changes in out-of-distribution data compared to changes that more dramatically change the statistical properties of the images, such as nonstandard backgrounds. Further validation on additional datasets (particularly from institutions with different photographic and tissue-handling practices) will be important to assess and potentially improve the generalizability of the tool.

Comment 2: *Qualitative Analysis of Segmentation Outliers*
The study notes that 6% of the out-of-distribution UW-fixed images exhibited errors (mean surface distance) greater than 2 mm.
Suggestion: I recommend adding a brief qualitative analysis or a supplementary figure illustrating these outlier cases. Identifying if these errors were driven by tissue artifacts, excessive glare, or poor contrast between the cortex and the background would help future users understand the specific conditions where manual refinement is most likely needed.

The discussion section of the revised manuscript illustrates what conditions typically lead to worse performance, including imperfect manual annotations for out of distribution photographs. The new Figure 7 shows a representative image from the worst performing photographs in the dataset and how it compares to the ground truth segmentation. Additionally, we added the following at the end of the Experiments and results section:

> To determine modes of failure, we examined the worst performing images in our out-of-distribution data. Among the worst performing images, the main driver for error came from ambiguous regions that resulted in high variability in manual annotations. This occurred most frequently along the pial surface (Figure 7), where the resulting discrepancies were often minor and could be corrected efficiently when very high accuracy was required.

Comment 3: *Currently, for the MADRC and UW-fixed datasets, pixel size calibration still requires manual clicks on reference rulers. In contrast, the UW-fresh dataset utilizes an automated detection of fiducial markers. Suggestion: The authors should discuss the feasibility of automating ruler detection for the other protocols. This would move the pipeline toward being fully autonomous, further reducing the "laborious manual intervention" mentioned in your abstract.*

We appreciate the idea of further automating the tool, including automation of pixel correction for processing retrospective data. In the discussion section we mention full automation as potential future work to further automate data processing, while discussing potential challenges with generalizability due to different reference rulers or scale bars and the possibility of falling back to manual clicking, if a user is unhappy with the results.

> Future work will explore how other parts of the preprocessing process can be automated. For example, the current need for manual correction of retrospective data with be circumvented via automatic detection of reference landmarks (e.g., rulers), while keeping the opportunity for manual editing when needed.

Comment 4: *Refining the Handling of Non-Target Slabs*
You mention that the presence of non-target slabs in the background can lead to irrelevant false positives which are currently factored out of accuracy metrics.
Suggestion: To improve the "out-of-the-box" user experience of the public tool, please clarify if the released version includes an automated post-processing step (such as a "largest connected component" filter) to remove these artifacts automatically, or if users must still perform this masking manually.

In the introduction section we added more information and Figure 1 to discuss the other steps of the tool, like manual selection of connected components after binary segmentation. The introduction section now includes and outline of the whole processing pipeline of the public tool.

> Prior to 3D reconstruction, gross tissue photographs must be preprocessed. First, all photographs must be corrected for pixel size and, ideally, perspective. This ensures that every pixel corresponds to a square of the same size. Next, each slab face must be segmented (often manually) to isolate them from the background and to remove any visible pial surface outside the cut plane. Finally, the slab order and connected components must be defined, critical for disjoint tissue slabs, commonly seen in slabs which include the temporal pole. We have made tools available to facilitate image preprocessing using a graphical user interface created specifically for this task (*surfer.nmr.mgh.harvard.edu/fswiki/PhotoTools*).

Additionally, we clarify within the results section and Figure 4, where we can see irrelevant slabs, how the next step in the processing pipeline deals with these false positives.

> This is to reflect the fact that we do not mind false positives far away from the target slabs (e.g., due to the presence of non-target slabs, as in Figure 5f or Figures S3 and S4 in the supplement), and which can be easily masked during postprocessing. In Figure 4, we show how users can select connected components using the graphical user interface on our publicly available tools, easily omitting any false positives in model predictions.

Comment 5: Data Availability and Repository Maintenance
As a requirement for PLOS One, all data underlying the findings must be fully available.
Suggestion: Ensure that the provided link to the FreeSurfer software suite and the specific tool repository is

active and that any necessary documentation for external users to run the model on their own data is clearly outlined.

We thank the reviewer for their time and expertise to further improve the manuscript. We would like to mention that, all training data is now fully available through Zenodo (https://zenodo.org/records/20647553), and will be liked within our public documentation, which outlines a tutorial to how to use the tool in the current version of Freesurfer.

Response to Reviewer 2

Comment 1: *Please clarify the research gap further in the abstract.*

Although segmentation tools have used in many pathology contexts, our approach focuses on the availability of an open-source tool, and data availability of manual segmentations. We have edited the abstract to further emphasize the motivation of the work through the needs that the tool is addressing.

> One caveat of this methodology is the requirement of segmentation of the tissue from the background and out-of-slice tissue in photographs, which currently requires laborious manual intervention. Manual delineation is a bottleneck in this process and poses challenges in scalability, and resources, restricting adoption of these methods.

Comment 2: *Rewrite the contributions as clear and concise bullet points.*

We have edited the Contribution section to summarize the contributions of the method in concise bullet points

In summary the main contribution of this work are:

- We share with the research community a segmentation tool of neuropathology brain slabs as part of the FreeSurfer Photo-Tools software suite *surfer.nmr.mgh.harvard.edu/fswiki/PhotoTools*, facilitating quantitative analysis of *post mortem* tissue slabs.
- We present performance evaluation of the tool comparing it with intra- and inter- rater variability of human labelers. Demonstrating that our tool reaches performance comparable of humans with a median Dice score over 0.98, mean surface distance under 0.4 mm, and 95% Hausdorff distance under 1.60 mm.
- We share a unique dataset of 1,573 manually labeled brain slab images from two different sites, spanning fresh and fixed tissue. Providing useful training or validation data for future segmentation models. Available at *zenodo.org/records/20647553*.

Comment 3: *Add a table summarizing related works, including the year of publication, the dataset used, the advantages and disadvantages of each research paper, and the nature of the technology used.*

To the best of our knowledge, this is the first tool for segmenting full brain tissue slabs in routine dissection photography; we have clarified this in the revised manuscript.

Comment 4: *Add the conclusions reached in the manuscript along with the proposed future works.*

We added an additional information to the Discussion section summarizing the key findings, including that automatic segmentation tools make volumetric analysis of dissection photographs more feasible, speeding up data preprocessing, and that a segmentation model can be generalizable sufficiently to out-of-distribution images from different sites and photographic setups.

Comment 5: *Add at least three references published in 2025*

We included references published in 2025 or newer, including current segmentation tools in different medical imaging context, and specifically pathology imaging.

18. Gopinath K, Greve D, Das S, Arnold S, Magdamo C, Iglesias JE. Cortical analysis of heterogeneous clinical brain MRI scans for large-scale neuroimaging studies. International Conference on Medical Image Computing and Computer-Assisted Intervention 2023;:35-45.

19. Aguirre M, Williams-Ramirez J, Zemlyanker D, Hu X, Deden-Binder L, Herisse R et al. Improving Neuropathological Reconstruction Fidelity via AI Slice Imputation. arXiv preprint arXiv:2602.00669 2026;.

21. Athalye C, Bahena A, Khandelwal P, Emrani S, Trotman W, Levorse L, et al. Operationalizing postmortem pathology-MRI association studies in Alzheimer's disease and related disorders with MRI-guided histology sampling. Acta Neuropathologica Communications. 2025;13(1):120

40. Chen J, Lu Y, Yu Q, Luo X, Adeli E, Wang Y, et al. Transunet: Transformers make strong encoders for medical image segmentation. arXiv preprint arXiv:2102.04306. 2021;.

41. Li J, Chen J, Jiang L, Li R, Han P, Cheng J. SEAformer: Selective Edge Aggregation transformer for 2D medical image segmentation. Biomedical Signal Processing and Control. 2025;102:107203.

46. Ma J, Guo Z, Zhou F, Wang Y,Xu Y, Li J, et al. A generalizable pathology foundation model using a unified knowledge distillation pretraining framework. Nature Biomedical Engineering. 2026;10(3):545-564

47. Wu, J, Ji W, Fu H, Xu M, Jin Y, Xu Y. Medsegdiff-v2: Diffusion-based medical image segmentation with transformer. Proceedings of the AAAI conference on artificial intelligence. 2024;38(6):6030-6038

48. Xing Z, Wan L, Fu H, Yang G, Yang Y, Yu L, et al. Diff-UNet: A diffusion embedded network for robust 3D medical image segmentation. Medical Image Analysis. 2025;105:103654

Comment 6:*Add a flowchart summarizing the proposed method.*

Figure 1 in the revised version is new and outlines the steps for preprocessing images for 3D analysis, highlighting the role of our new segmentation tool in this process.

Response to Editor Comments

Comment 1: *Please ensure that your manuscript meets PLOSONE's style requirements, including those for file naming. The PLOS ONE style templates can be found at https://journals.plos.org/plosone/s/file?id=wjVg/PLOSOne_formatting_sample_main_body.pdf and journals.plos.org/plosone/s/file?id=ba62/PLOSOne_formatting_sample_title_authors_affiliations.pdf.*

We have reviewed the style requirements provided.

Comment 2: *Please note that PLOS One has specific guidelines on code sharing for submissions in which author-generated code underpins the findings in the manuscript. In these cases, all author-generated code must be made available without restrictions upon publication of the work. Please review our guidelines at https://journals.plos.org/plosone/s/materials-and-software-sharing#loc-sharing-code and ensure that your code is shared in a way that follows best practice and facilitates reproducibility and reuse.*

We reviewed the code sharing guidelines. All model training was done using the open source tool nnUNet [https://github.com/mic-dkfz/nnunet]. No custom code was created for this study, therefore the code sharing guidelines are not applicable.

Comment 3*: Thank you for stating in your Funding Statement:*

"This research was primarily funded by the National Institute of Aging, https://www.nia.nih.gov/, (1R01AG070988 (JEI)). Additional support was provided by the National Institute of Mental Health, https://www.nimh.nih.gov/, (1RF1MH123195 (JEI, BF), 1UM1MH130981), the National Institute of Aging,https://www.nia.nih.gov/, (1RF1AG080371 (JEI)), the National Institute of Biomedical Imaging and Bioengineering, https://www.nibib.nih.gov/, (1R01EB031114), and the National Institute of Neurological Disorders and Stroke, https://www.ninds.nih.gov/, (1R21NS138995). The UW BioRepository and Integrated Neuropathology (BRaIN) laboratory is supported by the National Institutes of Health (NIH), by the National Institute of Mental Health, https://www.nimh.nih.gov/, through the the BRAIN Initiative Cell Atlas Network (UM1MH134812 (CDK)); also by the National Institute of Aging, https://www.nia.nih.gov/, through the UW Alzheimer's Disease Research Center (P30 AG066509 (CDK)), the Kaiser Permanente Washington Adult Changes in Thought (ACT) study (U19 AG066567 (CDK)), the Seattle Alzheimer's Disease Brain Cell Atlas (U19AG060909 (CDK)), also multiple collaborative projects supporting imaging, digital pathology, neuropathology, and related research tools (U24AG072458 (CDK); through the National Institute of Neurological Disorders and Stroke, https://www.ninds.nih.gov/, U24NS133949; U24NS133945; U24NS135651; U01NS137500; and U01NS137484 (CDK)), the US Department of Defense, https://www.war.gov/, (DoD W81XWH-21-S- TBIPH2), the Allen Institute for Brain Science, and the Nancy and Buster Alvord Endowment (to CDK). Support for this research was also provided in part by the BRAIN Initiative Cell Atlas Network (BICAN) grants, through the National Institute of Mental Health, https://www.nimh.nih.gov/, U01MH117023 (BF), UM1MH134812 (CDK) and UM1MH130981, the Brain Initiative Brain Connects consortium, through the National Institute of Neurological Disorders and Stroke, https://www.ninds.nih.gov/, (U01NS132181 (BF), 1UM1NS132358-01), the National Institute for Biomedical Imaging and Bioengineering, https://www.nibib.nih.gov/, (1R01EB023281 (BF), R21EB018907 (BF), R01EB019956 (BF), P41EB030006 (BF)), the National Institute on Aging, https://www.nia.nih.gov/, (R21AG082082 (BF), 1R01AG064027 (BF), R01AG016495, 1R01AG070988 (JEI)), the National Institute of Mental Health, https://www.nimh.nih.gov/, (UM1MH130981, R01 MH123195 (BF, JEI), R01 MH121885, 1RF1MH123195 (BF, JEI)), the National Institute for Neurological Disorders and Stroke, https://www.ninds.nih.gov/, (1U24NS135561-01 (BF), R01NS070963 (BF), 2R01NS083534 (BF), R01NS105820 (BF), R25NS125599 (BF)), and was made possible by the resources provided by Shared Instrumentation Grants, through National Center for Research Resources, http://www.ncrr.nih.gov/, 1S10RR023401 (BF), 1S10RR019307 (BF), and 1S10RR023043. Additional support was provided by the NIH Blueprint for Neuroscience Research through National Institute of Mental Health, https://www.nimh.nih.gov/, (5U01-MH093765), part of the multi institutional Human Connectome Project. Much of the computation resources required for this research was performed on computational hardware generously provided by the Massachusetts Life Sciences Center (https://www.masslifesciences.com). The authors would like to thank the research participants and their families without whom this work would be impossible."

*Please provide an amended statement that declares *all* the funding or sources of support (whether external or internal to your organization) received during this study, as detailed online in our guide for authors at http://journals.plos.org/plosone/s/submit-now. Please also include the statement "There was no additional external funding received for this study." in your updated Funding Statement.*
Please include your amended Funding Statement within your cover letter. We will change the online submission form on your behalf.

We include an amended statement consistent with the disclosure of funding sources guidelines, and updated the statement to include that no other external funding was received for the study.

This research was primarily funded by the National Institute of Aging (1R01AG070988 (JEI)). Additional support was provided by the National Institute of Mental Health (1RF1MH123195 (JEI, BF), 1UM1MH130981), the National Institute of Aging (1RF1AG080371 (JEI)), the National Institute of Biomedical Imaging and Bioengineering,, (1R01EB031114), and the National Institute of Neurological Disorders and Stroke (1R21NS138995). The UW BioRepository and Integrated Neuropathology (BRaIN) laboratory is supported by the National Institutes of Health (NIH), by the National Institute of Mental Health, through the the BRAIN Initiative Cell Atlas Network (UM1MH134812 (CDK)); also by the National Institute of Aging through the UW Alzheimer's Disease Research Center (P30 AG066509 (CDK)), the Kaiser Permanente Washington Adult Changes in Thought (ACT) study (U19 AG066567 (CDK)), the Seattle Alzheimer's Disease Brain Cell Atlas (U19AG060909 (CDK)), also multiple collaborative projects supporting imaging, digital pathology, neuropathology, and related research tools (U24AG072458 (CDK); through the National Institute of Neurological Disorders and Stroke, U24NS133949; U24NS133945; U24NS135651; U01NS137500; and U01NS137484 (CDK)), the US Department of Defense (DoD W81XWH-21-S- TBIPH2), the Allen Institute for Brain Science, and the Nancy and Buster Alvord Endowment (to CDK). Support for this research was also provided in part by the BRAIN Initiative Cell Atlas Network (BICAN) grants, through the National Institute of Mental Health U01MH117023 (BF), UM1MH134812 (CDK) and UM1MH130981, the Brain Initiative Brain Connects consortium, through the National Institute of Neurological Disorders and Stroke (U01NS132181 (BF), 1UM1NS132358-01), the National Institute for Biomedical Imaging and Bioengineering (1R01EB023281 (BF), R21EB018907 (BF), R01EB019956 (BF), P41EB030006 (BF)), the National Institute on Aging, (R21AG082082 (BF), 1R01AG064027 (BF), R01AG016495, 1R01AG070988 (JEI)), the National Institute of Mental Health (UM1MH130981, R01 MH123195 (BF, JEI), R01 MH121885, 1RF1MH123195 (BF, JEI)), the National Institute for Neurological Disorders and Stroke (1U24NS135561-01 (BF), R01NS070963 (BF), 2R01NS083534 (BF), R01NS105820 (BF), R25NS125599 (BF)), and was made possible by the resources provided by Shared Instrumentation Grants, through National Center for Research Resources 1S10RR023401 (BF), 1S10RR019307 (BF), and 1S10RR023043. Additional support was provided by the NIH Blueprint for Neuroscience Research through National Institute of Mental Health (5U01-MH093765), part of the multi-institutional Human Connectome Project. Much of the computation resources required for this research was performed on computational hardware generously provided by the Massachusetts Life Sciences Center. The authors would like to thank the research participants and their families without whom this work would be impossible. The funders had no role in study design, data collection and analysis, decision to publish, or preparation of the manuscript. There was no additional external funding received for this study. The commercial affiliations declared in the competing interests statement provided support in the form of salaries for authors BH and BF, but did not have any additional role in the study design, data collection and analysis, decision to publish, or preparation of the manuscript.

Comment 4: *Thank you for stating the following in the Competing Interests section:*
"Dr Hyman owns stock in Novartis; he serves on the SAB of Dewpoint and has an option for stock. He serves on a scientific advisory board or is a consultant for AbbVie, Alexion, Ambagon, Aprinoia Therapeutics, Arbor Bio, Arvinas,

Avrobio, AstraZenica, Biogen, Bioinsights, BMS, Cell Signaling, Cure Alz Fund, CurieBio, Dewpoint, Etiome, Latus, Merck, Novartis, Paragon, Pfizer, Sanofi, Sofinnova, SV Health, Takeda, TD Cowen, Vigil, Violet, Voyager, WaveBreak. His laboratory is supported by research grants from the National Institutes of Health, Cure Alzheimer's Fund, Tau Consortium, and the JPB Foundation – and sponsored research agreement from Abbvie and Sanofi. He has a collaborative project with Biogen and Neurimmune. Dr Fischl is an advisor to DeepHealth, a company whose medical pursuits focus on medical imaging and measurement technologies. BF's interests were reviewed and are managed by Massachusetts General Hospital and Mass. General Brigham in accordance with their conflict of interest policies."

We note that one or more of the authors are employed by a commercial company:
a. Please provide an amended Funding Statement declaring this commercial affiliation, as well as a statement regarding the Role of Funders in your study. If the funding organization did not play a role in the study design, data collection and analysis, decision to publish, or preparation of the manuscript and only provided financial support in the form of authors' salaries and/or research materials, please review your statements relating to the author contributions, and ensure you have specifically and accurately indicated the role(s) that these authors had in your study. You can update author roles in the Author Contributions section of the online submission form.
Please also include the following statement within your amended Funding Statement.
"The funder provided support in the form of salaries for authors [insert relevant initials], but did not have any additional role in the study design, data collection and analysis, decision to publish, or preparation of the manuscript. The specific roles of these authors are articulated in the 'author contributions' section."
If your commercial affiliation did play a role in your study, please state and explain this role within your updated Funding Statement.
b. Please also provide an updated Competing Interests Statement declaring this commercial affiliation along with any other relevant declarations relating to employment, consultancy, patents, products in development, or marketed products, etc.
Within your Competing Interests Statement, please confirm that this commercial affiliation does not alter your adherence to all PLOS ONE policies on sharing data and materials by including the following statement: "This does not alter our adherence to PLOS ONE policies on sharing data and materials." (as detailed online in our guide for authors http://journals.plos.org/plosone/s/competing-interests) . If this adherence statement is not accurate and there are restrictions on sharing of data and/or materials, please state these. Please note that we cannot proceed with consideration of your article until this information has been declared.
Please include both an updated Funding Statement and Competing Interests Statement in your cover letter. We will change the online submission form on your behalf.

a. We include an amended statement detailing that outside commercial sources did not play a role in the funding of the study, nor the study itself. Within the funding statement we state:

   The commercial affiliations declared in the competing interests statement provided support in the form of salaries for authors BH and BF, but did not have any additional role in the study design, data collection and analysis, decision to publish, or preparation of the manuscript.

b. We updated a competing interest statement including declarations for all potential sources of conflict.

   Hyman owns stock in Novartis; he serves on the SAB of Dewpoint and has an option for stock. He serves on a scientific advisory board or is a consultant for AbbVie, Alexion, Ambagon, Aprinoia Therapeutics, Arbor Bio, Arvinas, Avrobio, AstraZenica, Biogen, Bioinsights, BMS, Cell Signaling, Cure Alz Fund, CurieBio, Dewpoint, Etiome, Latus, Merck, Novartis, Paragon, Pfizer, Sanofi, Sofinnova, SV Health, Takeda, TD Cowen, Vigil, Violet, Voyager, WaveBreak. His laboratory is supported by research grants from the National Institutes of Health, Cure Alzheimer's Fund, Tau Consortium, and the JPB Foundation

– and sponsored research agreement from Abbvie and Sanofi. He has a collaborative project with Biogen and Neurimmune. Dr Fischl is an advisor to DeepHealth, a company whose medical pursuits focus on medical imaging and measurement technologies. BF's interests were reviewed and are managed by Massachusetts General Hospital and Mass. General Brigham in accordance with their conflict of interest policies. These affiliations did not play a role in the study design, data collection and analysis, decision to publish, or preparation of the manuscript and only provided financial support in the form of authors' salaries (BH, BF). These affiliations do not alter our adherence to PLOS ONE policies on sharing data and materials.

Comment 5: *When completing the data availability statement of the submission form, you indicated that you will make your data available on acceptance. We strongly recommend all authors decide on a data sharing plan before acceptance, as the process can be lengthy and hold up publication timelines. Please note that, though access restrictions are acceptable now, your entire data will need to be made freely accessible if your manuscript is accepted for publication. This policy applies to all data except where public deposition would breach compliance with the protocol approved by your research ethics board. If you are unable to adhere to our open data policy, please kindly revise your statement to explain your reasoning and we will seek the editor's input on an exemption. Please be assured that, once you have provided your new statement, the assessment of your exemption will not hold up the peer review process.*

We have all training data available through Zenodo, *zenodo.org/records/20647553*, including all training images and manually annotated labels.

Comment 6: *Please be informed that funding information should not appear in the Acknowledgments section or other areas of your manuscript. We will only publish funding information present in the Funding Statement section of the online submission form. Please remove any funding-related text from the manuscript.*

We have removed all mentions of funding from the acknowledgement sections.

Comment 7:*If the reviewer comments include a recommendation to cite specific previously published works, please review and evaluate these publications to determine whether they are relevant and should be cited. There is no requirement to cite these works unless the editor has indicated otherwise.*

We noted that no specific publications were recommended by the reviewer comments for citation.

The Athinoula A. Martinos Center for Biomedical Imaging @ Massachusetts General Hospital
149 13th Street, Suite 2301 | Charlestown, MA 02129
