# Supplementary material for: A public generalizable AI tool for automated segmentation of coronal brain tissue slabs for 3D neuropathology
Source: PLoS One. 2026 Aug 17;21(8):e0355740. doi: 10.1371/journal.pone.0355740 (PMC13480592; doi:10.1371/journal.pone.0355740)
Supplement: Peer review history

# Peer review: Decision Letter 0

**DOI:** 10.1371/journal.pone.0355740.r001

**Authors:** Federico Giove

24 Apr 2026

PONE-D-25-68120

A public generalizable AI tool for automated segmentation of coronal brain tissue slabs for 3D neuropathology

PLOS One

Dear Dr. Williams Ramirez,

Thank you for submitting your manuscript to PLOS ONE. After careful consideration, we feel that it has merit but does not fully meet PLOS ONE’s publication criteria as it currently stands. Therefore, we invite you to submit a revised version of the manuscript that addresses the points raised during the review process.

Please follow the indications of reviewers, in particualr reviewer #1

Please submit your revised manuscript by Jun 08 2026 11:59PM. If you will need more time than this to complete your revisions, please reply to this message or contact the journal office at plosone@plos.org. When you're ready to submit your revision, log on to https://www.editorialmanager.com/pone/ and select the 'Submissions Needing Revision' folder to locate your manuscript file.

Please include the following items when submitting your revised manuscript:

- A letter that responds to each point raised by the academic editor and reviewer(s). You should upload this letter as a separate file labeled 'Response to Reviewers'.
- A marked-up copy of your manuscript that highlights changes made to the original version. You should upload this as a separate file labeled 'Revised Manuscript with Track Changes'.
- An unmarked version of your revised paper without tracked changes. You should upload this as a separate file labeled 'Manuscript'.

If you would like to make changes to your financial disclosure, please include your updated statement in your cover letter. Guidelines for resubmitting your figure files are available below the reviewer comments at the end of this letter.

If applicable, we recommend that you deposit your laboratory protocols in protocols.io to enhance the reproducibility of your results. Protocols.io assigns your protocol its own identifier (DOI) so that it can be cited independently in the future. For instructions see: https://journals.plos.org/plosone/s/submission-guidelines#loc-laboratory-protocols. Additionally, PLOS ONE offers an option for publishing peer-reviewed Lab Protocol articles, which describe protocols hosted on protocols.io. Read more information on sharing protocols at https://plos.org/protocols?utm_medium=editorial-email&utm_source=authorletters&utm_campaign=protocols.

As the corresponding author, your ORCID iD is verified in the submission system and will appear in the published article. PLOS supports the use of ORCID, and we encourage all coauthors to register for an ORCID iD and use it as well. Please encourage your coauthors to verify their ORCID iD within the submission system before final acceptance, as unverified ORCID iDs will not appear in the published article. *Only*  the individual author can complete the verification step; PLOS staff *cannot*  verify ORCID iDs on behalf of authors.

We look forward to receiving your revised manuscript.

Kind regards,

Federico Giove, PhD

Academic Editor

PLOS One

Journal Requirements:

When submitting your revision, we need you to address these additional requirements.

1. Please ensure that your manuscript meets PLOS ONE's style requirements, including those for file naming. The PLOS ONE style templates can be found at

https://journals.plos.org/plosone/s/file?id=wjVg/PLOSOne_formatting_sample_main_body.pdf and

https://journals.plos.org/plosone/s/file?id=ba62/PLOSOne_formatting_sample_title_authors_affiliations.pdf.

2. Please note that PLOS One has specific guidelines on code sharing for submissions in which author-generated code underpins the findings in the manuscript. In these cases, all author-generated code must be made available without restrictions upon publication of the work. Please review our guidelines at https://journals.plos.org/plosone/s/materials-and-software-sharing#loc-sharing-code and ensure that your code is shared in a way that follows best practice and facilitates reproducibility and reuse.

3. Thank you for stating in your Funding Statement:

“This research was primarily funded by the National Institute of Aging, https://www.nia.nih.gov/, (1R01AG070988 (JEI)). Additional support was provided by the National Institute of Mental Health, https://www.nimh.nih.gov/, (1RF1MH123195 (JEI, BF), 1UM1MH130981), the National Institute of Aging, https://www.nia.nih.gov/, (1RF1AG080371 (JEI)), the National Institute of Biomedical Imaging and Bioengineering, https://www.nibib.nih.gov/, (1R01EB031114), and the National Institute of Neurological Disorders and Stroke, https://www.ninds.nih.gov/, (1R21NS138995). The UW BioRepository and Integrated Neuropathology (BRaIN) laboratory is supported by the National Institutes of Health (NIH), by the National Institute of Mental Health, https://www.nimh.nih.gov/, through the the BRAIN Initiative Cell Atlas Network (UM1MH134812 (CDK)); also by the National Institute of Aging, https://www.nia.nih.gov/, through the UW Alzheimer’s Disease Research Center (P30 AG066509 (CDK)), the Kaiser Permanente Washington Adult Changes in Thought (ACT) study (U19 AG066567 (CDK)), the Seattle Alzheimer’s Disease Brain Cell Atlas (U19AG060909 (CDK)), also multiple collaborative projects supporting imaging, digital pathology, neuropathology, and related research tools (U24AG072458 (CDK); through the National Institute of Neurological Disorders and Stroke, https://www.ninds.nih.gov/, U24NS133949; U24NS133945; U24NS135651; U01NS137500; and U01NS137484 (CDK)), the US Department of Defense, https://www.war.gov/, (DoD W81XWH-21-S- TBIPH2), the Allen Institute for Brain Science, and the Nancy and Buster Alvord Endowment (to CDK). Support for this research was also provided in part by the BRAIN Initiative Cell Atlas Network (BICAN) grants, through the National Institute of Mental Health, https://www.nimh.nih.gov/, U01MH117023 (BF), UM1MH134812 (CDK) and UM1MH130981, the Brain Initiative Brain Connects consortium, through the National Institute of Neurological Disorders and Stroke, https://www.ninds.nih.gov/, (U01NS132181 (BF), 1UM1NS132358-01), the National Institute for Biomedical Imaging and Bioengineering, https://www.nibib.nih.gov/, (1R01EB023281 (BF), R21EB018907 (BF), R01EB019956 (BF), P41EB030006 (BF)), the National Institute on Aging, https://www.nia.nih.gov/, (R21AG082082 (BF), 1R01AG064027 (BF), R01AG016495, 1R01AG070988 (JEI)), the National Institute of Mental Health, https://www.nimh.nih.gov/, (UM1MH130981, R01 MH123195 (BF, JEI), R01 MH121885, 1RF1MH123195 (BF, JEI)), the National Institute for Neurological Disorders and Stroke, https://www.ninds.nih.gov/, (1U24NS135561-01 (BF), R01NS070963 (BF), 2R01NS083534 (BF), R01NS105820 (BF), R25NS125599 (BF)), and was made possible by the resources provided by Shared Instrumentation Grants, through National Center for Research Resources, http://www.ncrr.nih.gov/, 1S10RR023401 (BF), 1S10RR019307 (BF), and 1S10RR023043. Additional support was provided by the NIH Blueprint for Neuroscience Research through National Institute of Mental Health, https://www.nimh.nih.gov/, (5U01-MH093765), part of the multi institutional Human Connectome Project. Much of the computation resources required for this research was performed on computational hardware generously provided by the Massachusetts Life Sciences Center (https://www.masslifesciences.com). The authors would like to thank the research participants and their families without whom this work would be impossible. “

Please provide an amended statement that declares *all* the funding or sources of support (whether external or internal to your organization) received during this study, as detailed online in our guide for authors at http://journals.plos.org/plosone/s/submit-now.  Please also include the statement “There was no additional external funding received for this study.” in your updated Funding Statement.

Please include your amended Funding Statement within your cover letter. We will change the online submission form on your behalf.

4. Thank you for stating the following in the Competing Interests section:

“Dr Hyman owns stock in Novartis; he serves on the SAB of Dewpoint and has an option for stock. He serves on a scientific advisory board or is a consultant for AbbVie, Alexion, Ambagon, Aprinoia Therapeutics, Arbor Bio, Arvinas, Avrobio, AstraZenica, Biogen, Bioinsights, BMS, Cell Signaling, Cure Alz Fund, CurieBio, Dewpoint, Etiome, Latus, Merck, Novartis, Paragon, Pfizer, Sanofi, Sofinnova, SV Health, Takeda, TD Cowen, Vigil, Violet, Voyager, WaveBreak. His laboratory is supported by research grants from the National Institutes of Health, Cure Alzheimer’s Fund, Tau Consortium, and the JPB Foundation – and sponsored research agreement from Abbvie and Sanofi. He has a collaborative project with Biogen and Neurimmune. Dr Fischl is an advisor to DeepHealth, a company whose medical pursuits focus on medical imaging and measurement technologies. BF's interests were reviewed and are managed by Massachusetts General Hospital and Mass. General Brigham in accordance with their conflict of interest policies. “

We note that one or more of the authors are employed by a commercial company:

a.        Please provide an amended Funding Statement declaring this commercial affiliation, as well as a statement regarding the Role of Funders in your study. If the funding organization did not play a role in the study design, data collection and analysis, decision to publish, or preparation of the manuscript and only provided financial support in the form of authors' salaries and/or research materials, please review your statements relating to the author contributions, and ensure you have specifically and accurately indicated the role(s) that these authors had in your study. You can update author roles in the Author Contributions section of the online submission form.

Please also include the following statement within your amended Funding Statement.

“The funder provided support in the form of salaries for authors [insert relevant initials], but did not have any additional role in the study design, data collection and analysis, decision to publish, or preparation of the manuscript. The specific roles of these authors are articulated in the ‘author contributions’ section.”

If your commercial affiliation did play a role in your study, please state and explain this role within your updated Funding Statement.

b. Please also provide an updated Competing Interests Statement declaring this commercial affiliation along with any other relevant declarations relating to employment, consultancy, patents, products in development, or marketed products, etc.

Within your Competing Interests Statement, please confirm that this commercial affiliation does not alter your adherence to all PLOS ONE policies on sharing data and materials by including the following statement: "This does not alter our adherence to  PLOS ONE policies on sharing data and materials.” (as detailed online in our guide for authors http://journals.plos.org/plosone/s/competing-interests) . If this adherence statement is not accurate and  there are restrictions on sharing of data and/or materials, please state these. Please note that we cannot proceed with consideration of your article until this information has been declared.

Please include both an updated Funding Statement and Competing Interests Statement in your cover letter. We will change the online submission form on your behalf.

5. When completing the data availability statement of the submission form, you indicated that you will make your data available on acceptance. We strongly recommend all authors decide on a data sharing plan before acceptance, as the process can be lengthy and hold up publication timelines. Please note that, though access restrictions are acceptable now, your entire data will need to be made freely accessible if your manuscript is accepted for publication. This policy applies to all data except where public deposition would breach compliance with the protocol approved by your research ethics board. If you are unable to adhere to our open data policy, please kindly revise your statement to explain your reasoning and we will seek the editor's input on an exemption. Please be assured that, once you have provided your new statement, the assessment of your exemption will not hold up the peer review process.

6. Please be informed that funding information should not appear in the Acknowledgments section or other areas of your manuscript. We will only publish funding information present in the Funding Statement section of the online submission form. Please remove any funding-related text from the manuscript.

7. If the reviewer comments include a recommendation to cite specific previously published works, please review and evaluate these publications to determine whether they are relevant and should be cited. There is no requirement to cite these works unless the editor has indicated otherwise.

[Note: HTML markup is below. Please do not edit.]

Reviewers' comments:

Reviewer's Responses to Questions

**Comments to the Author**

1. Is the manuscript technically sound, and do the data support the conclusions?

The manuscript must describe a technically sound piece of scientific research with data that supports the conclusions. Experiments must have been conducted rigorously, with appropriate controls, replication, and sample sizes. The conclusions must be drawn appropriately based on the data presented.

Reviewer #1: Yes

Reviewer #2: Yes

**********

2. Has the statistical analysis been performed appropriately and rigorously?

Reviewer #1: I Don't Know

Reviewer #2: Yes

**********

3. Have the authors made all data underlying the findings in their manuscript fully available?

The PLOS Data policy requires authors to make all data underlying the findings described in their manuscript fully available without restriction, with rare exception (please refer to the Data Availability Statement in the manuscript PDF file). The data should be provided as part of the manuscript or its supporting information, or deposited to a public repository. For example, in addition to summary statistics, the data points behind means, medians and variance measures should be available. If there are restrictions on publicly sharing data—e.g. participant privacy or use of data from a third party—those must be specified.

Reviewer #1: Yes

Reviewer #2: Yes

**********

4. Is the manuscript presented in an intelligible fashion and written in standard English?

PLOS ONE does not copyedit accepted manuscripts, so the language in submitted articles must be clear, correct, and unambiguous. Any typographical or grammatical errors should be corrected at revision, so please note any specific errors here.

Reviewer #1: Yes

Reviewer #2: Yes

**********

5. Review Comments to the Author

Please use the space provided to explain your answers to the questions above. You may also include additional comments for the author, including concerns about dual publication, research ethics, or publication ethics. (Please upload your review as an attachment if it exceeds 20,000 characters)

Reviewer #1: Below is a set of suggestions and recommendations for the improvement of your manuscript, "A public generalizable AI tool for automated segmentation of coronal brain tissue slabs for 3D neuropathology". This work addresses a critical bottleneck in quantitative neuropathology by automating the time-consuming process of manual tissue segmentation. Your results, achieving a median Dice score over 0.98 and performance approaching inter-/intra-rater levels, are highly impressive.

To further strengthen the manuscript for publication in PLOS One, please consider the following points:

1. Elaborate on Generalization and Site Diversity

While the model demonstrates strong robustness on out-of-distribution data, you correctly identify that data diversity is a limitation, as training and evaluation involved only three sites.

Suggestion: It would be beneficial to expand the discussion on how the tool might perform in more "extreme" out-of-distribution scenarios, such as varying lighting conditions, non-standard backgrounds, or different camera angles. Additionally, explicitly recommending future external validation by independent institutions with different photographic protocols would provide a clearer roadmap for the tool's adoption.

2. Qualitative Analysis of Segmentation Outliers

The study notes that 6% of the out-of-distribution UW-fixed images exhibited errors (mean surface distance) greater than 2 mm.

Suggestion: I recommend adding a brief qualitative analysis or a supplementary figure illustrating these outlier cases. Identifying if these errors were driven by tissue artifacts, excessive glare, or poor contrast between the cortex and the background would help future users understand the specific conditions where manual refinement is most likely needed.

3. Towards Full Automation of Calibration

Currently, for the MADRC and UW-fixed datasets, pixel size calibration still requires manual clicks on reference rulers. In contrast, the UW-fresh dataset utilizes an automated detection of fiducial markers.

Suggestion: The authors should discuss the feasibility of automating ruler detection for the other protocols. This would move the pipeline toward being fully autonomous, further reducing the "laborious manual intervention" mentioned in your abstract.

4. Refining the Handling of Non-Target Slabs

You mention that the presence of non-target slabs in the background can lead to irrelevant false positives which are currently factored out of accuracy metrics.

Suggestion: To improve the "out-of-the-box" user experience of the public tool, please clarify if the released version includes an automated post-processing step (such as a "largest connected component" filter) to remove these artifacts automatically, or if users must still perform this masking manually.

5. Data Availability and Repository Maintenance

As a requirement for PLOS One, all data underlying the findings must be fully available.

Suggestion: Ensure that the provided link to the FreeSurfer software suite and the specific tool repository is active and that any necessary documentation for external users to run the model on their own data is clearly outlined.

Conclusion: This is a technically sound and well-written manuscript that provides a valuable open-source contribution to the field. Addressing the points above will enhance the transparency regarding the tool's limitations and its practical utility for the broader neuropathology community.

Reviewer #2: 1- Please clarify the research gap further in the abstract.

2- Rewrite the contributions as clear and concise bullet points.

3- Add a table summarizing related works, including the year of publication, the dataset used, the advantages and disadvantages of each research paper, and the nature of the technology used.

4- Add the conclusions reached in the manuscript along with the proposed future works.

5- Add at least three references published in 2025

6-Add a flowchart summarizing the proposed method.

**********

6. PLOS authors have the option to publish the peer review history of their article (what does this mean?). If published, this will include your full peer review and any attached files.

If you choose “no”, your identity will remain anonymous but your review may still be made public.

**Do you want your identity to be public for this peer review?**  For information about this choice, including consent withdrawal, please see our Privacy Policy.

Reviewer #1: No

Reviewer #2: No

**********

[NOTE: If reviewer comments were submitted as an attachment file, they will be attached to this email and accessible via the submission site. Please log into your account, locate the manuscript record, and check for the action link "View Attachments". If this link does not appear, there are no attachment files.]

To ensure your figures meet our technical requirements, please review our figure guidelines: https://journals.plos.org/plosone/s/figures

You may also use PLOS’s free figure tool, NAAS, to help you prepare publication quality figures: https://journals.plos.org/plosone/s/figures#loc-tools-for-figure-preparation.

NAAS will assess whether your figures meet our technical requirements by comparing each figure against our figure specifications.

# Author comment: Author response to Decision Letter 1

**DOI:** 10.1371/journal.pone.0355740.r002

2 Jul 2026

Dear editor and reviewers,

We would like to thank the editor and reviewers for their time and insightful feedback on our submission “A public generalizable AI tool for automated segmentation of coronal brain tissue slabs for 3D neuropathology.” We have carefully reviewed all comments and made appropriate changes to our manuscript.

Specifically, we have revised the manuscript to provide a clearer and more comprehensive account of the study’s methodology, context, and practical implications. We expanded the discussion of the image processing workflow and open-source tools, strengthened the manuscript’s connection to prior work on segmentation methods, and clarified how our model may be applied to neuropathology image analysis. We also added further evaluation of model performance in challenging out-of-distribution cases, including visual examples of disagreement between predictions and manual annotations. Together, these revisions improve the manuscript’s technical transparency, situate the work more fully within the existing literature, and provide clearer guidance for future development and end-user application.

Attached are our responses to all comments point-by-point, additionally to a revised manuscript with all changes highlighted in red.

Sincerely,

Jonathan Williams Ramirez, on behalf of the authors

Attachment Submitted filename: PLOSONE_response_20260702.pdf

# Peer review: Decision Letter 1

**DOI:** 10.1371/journal.pone.0355740.r003

**Authors:** Federico Giove

26 Jul 2026

A public generalizable AI tool for automated segmentation of coronal brain tissue slabs for 3D neuropathology

PONE-D-25-68120R1

Dear Dr. Williams Ramirez,

We’re pleased to inform you that your manuscript has been judged scientifically suitable for publication and will be formally accepted for publication once it meets all outstanding technical requirements.

Within one week, you’ll receive an e-mail detailing the required amendments. When these have been addressed, you’ll receive a formal acceptance letter and your manuscript will be scheduled for publication.

An invoice will be generated when your article is formally accepted. Please note, if your institution has a publishing partnership with PLOS and your article meets the relevant criteria, all or part of your publication costs will be covered. Please make sure your user information is up-to-date by logging into Editorial Manager at Editorial Manager® and clicking the ‘Update My Information' link at the top of the page. For questions related to billing, please contact billing support.

If your institution or institutions have a press office, please notify them about your upcoming paper to help maximize its impact. If they’ll be preparing press materials, please inform our press team as soon as possible -- no later than 48 hours after receiving the formal acceptance. Your manuscript will remain under strict press embargo until 2 pm Eastern Time on the date of publication. For more information, please contact onepress@plos.org.

Kind regards,

Federico Giove, PhD

Academic Editor

PLOS One

Additional Editor Comments (optional):

Reviewers' comments:

Reviewer's Responses to Questions

**Comments to the Author**

1. If the authors have adequately addressed your comments raised in a previous round of review and you feel that this manuscript is now acceptable for publication, you may indicate that here to bypass the “Comments to the Author” section, enter your conflict of interest statement in the “Confidential to Editor” section, and submit your "Accept" recommendation.

Reviewer #1: All comments have been addressed

Reviewer #2: (No Response)

**********

2. Is the manuscript technically sound, and do the data support the conclusions?

The manuscript must describe a technically sound piece of scientific research with data that supports the conclusions. Experiments must have been conducted rigorously, with appropriate controls, replication, and sample sizes. The conclusions must be drawn appropriately based on the data presented.

Reviewer #1: Yes

Reviewer #2: (No Response)

**********

3. Has the statistical analysis been performed appropriately and rigorously?

Reviewer #1: Yes

Reviewer #2: (No Response)

**********

4. Have the authors made all data underlying the findings in their manuscript fully available?

The PLOS Data policy requires authors to make all data underlying the findings described in their manuscript fully available without restriction, with rare exception (please refer to the Data Availability Statement in the manuscript PDF file). The data should be provided as part of the manuscript or its supporting information, or deposited to a public repository. For example, in addition to summary statistics, the data points behind means, medians and variance measures should be available. If there are restrictions on publicly sharing data—e.g. participant privacy or use of data from a third party—those must be specified.

Reviewer #1: Yes

Reviewer #2: (No Response)

**********

5. Is the manuscript presented in an intelligible fashion and written in standard English?

PLOS ONE does not copyedit accepted manuscripts, so the language in submitted articles must be clear, correct, and unambiguous. Any typographical or grammatical errors should be corrected at revision, so please note any specific errors here.

Reviewer #1: Yes

Reviewer #2: (No Response)

**********

6. Review Comments to the Author

Please use the space provided to explain your answers to the questions above. You may also include additional comments for the author, including concerns about dual publication, research ethics, or publication ethics. (Please upload your review as an attachment if it exceeds 20,000 characters)

Reviewer #1: (No Response)

Reviewer #2: (No Response)

**********

7. PLOS authors have the option to publish the peer review history of their article (what does this mean?). If published, this will include your full peer review and any attached files.

If you choose “no”, your identity will remain anonymous but your review may still be made public.

**Do you want your identity to be public for this peer review?**  For information about this choice, including consent withdrawal, please see our Privacy Policy.

Reviewer #1: No

Reviewer #2: No

**********

# Editor report: Acceptance letter

**DOI:** 10.1371/journal.pone.0355740.r004

**Authors:** Federico Giove

PONE-D-25-68120R1

PLOS One

Dear Dr. Williams Ramirez,

I'm pleased to inform you that your manuscript has been deemed suitable for publication in PLOS One. Congratulations! Your manuscript is now being handed over to our production team.

At this stage, our production department will prepare your paper for publication. This includes ensuring the following:

* All references, tables, and figures are properly cited

* All relevant supporting information is included in the manuscript submission,

* There are no issues that prevent the paper from being properly typeset

You will receive further instructions from the production team, including instructions on how to review your proof when it is ready. Please keep in mind that we are working through a large volume of accepted articles, so please give us a few days to review your paper and let you know the next and final steps.

Lastly, if your institution or institutions have a press office, please let them know about your upcoming paper now to help maximize its impact. If they'll be preparing press materials, please inform our press team within the next 48 hours. Your manuscript will remain under strict press embargo until 2 pm Eastern Time on the date of publication. For more information, please contact onepress@plos.org.

You will receive an invoice from PLOS for your publication fee after your manuscript has reached the completed accept phase. If you receive an email requesting payment before acceptance or for any other service, this may be a phishing scheme. Learn how to identify phishing emails and protect your accounts at https://explore.plos.org/phishing.

If we can help with anything else, please email us at customercare@plos.org.

Thank you for submitting your work to PLOS ONE and supporting open access.

Kind regards,

PLOS ONE Editorial Office Staff

on behalf of

Dr. Federico Giove

Academic Editor

PLOS One
